# CXCL11 secreted by cancer-associated fibroblasts promotes nasopharyngeal carcinoma progression via CXCR3/PD-L1 axis

**DOI:** 10.1080/19336918.2026.2665498

**Published:** 2026-05-04

**Authors:** Jie Fang, Huijuan Cheng, Peng Zhang, Liang Wang

**Affiliations:** Department of Head and Neck Surgery, The First Affiliated Hospital of Zhengzhou University, Zhengzhou, Henan Province, China

**Keywords:** Cancer-associated fibroblasts, CXCL11, CXCR3, nasopharyngeal carcinoma, PD-L1

## Abstract

The secretion of chemokines by cancer-associated fibroblasts (CAFs) is a critical driver of cancer progression. Nevertheless, the precise contribution of CAFs in the nasopharyngeal carcinoma (NPC) tumor microenvironment to disease progression is yet to be fully understood. In this study, C-X-C motif chemokine ligand 11 (CXCL11) was identified to be upregulated in tumor tissues of NPC patients and NPC cells compared to counterpart normal tissues and cell lines. The CAFs-secreted CXCL11 was found to enhance the proliferative, invasive, and migratory capacities of NPC cells. CAFs-derived CXCL11 upregulates CXCR3 expression to facilitate NPC cell proliferation, migration, and invasion. Through mechanism investigation, we confirmed that CXCL11/CXCR3 axis upregulated PD-L1 expression through p65-mediated transcription activation. Finally, *in vivo* experiments further validated the tumor-promoting role of CAFs-secreted CXCL11 in NPC. In conclusion, our findings reveal a novel mechanism wherein CAFs-secreted CXCL11 promotes NPC malignant progression by activating the CXCR3/PD-L1 signaling axis.

## Introduction

As a prevalent head and neck malignancy, nasopharyngeal carcinoma (NPC) develops from the epithelial lining of the nasopharynx [1]. The insidious nature of its early pathogenesis, coupled with a lack of specific symptoms, results in the majority of patients (over 70%) presenting with locally advanced disease at their initial diagnosis [2]. Although studies have been conducted to develop therapeutic methods for NPC, the oncogenic genomic changes during the progression of NPC remain far from clear. Therefore, investigating molecular mechanisms affecting the malignant progression of NPC is crucial to the development of more biomarkers or potential therapeutic markers.

As a pivotal component of the tumor microenvironment, cancer-associated fibroblasts (CAFs) are known to critically modulate cancer progression [3]. Importantly, CAFs can secrete chemokines such as CXC-chemokine family to accelerate the malignant progression of human cancers [4]. C-X-C motif chemokine ligand 11 (CXCL11) has been attested to exert oncogenic functions to aggravate the malignant progress of human cancers. For example, CXCL11 upregulated by LRPPRC-mediated m6A modification drives breast cancer cell malignancy [5]. CXCL11 is negatively regulated by YY1/miR-548t-5p axis and facilitates pancreatic cancer metastasis [6]. However, whether CXCL11 can be secreted by CAFs to regulate NPC cell malignancy has not been well investigated.

CXCR3 is a receptor for the chemokine CXCL11. Increased secretion of CXCL11 from donor cells is closely associated with elevated CXCR3 expression in recipient cells [7]. In an immune-active microenvironment, CXCL11 binds to CXCR3 to efficiently recruit cytotoxic T cells (CD8^+^ T cells) and natural killer cells (NK cells) – key anti-tumor effector cells – into the tumor, thereby initiating a potent immune response to eliminate cancer cells. However, in an immunosuppressive microenvironment, the CXCL11/CXCR3 axis has been reported to activate tumor-associated signaling pathways and contribute to the progression of head and neck tumors [8]. However, it remains unclear whether CAFs-derived CXCL11 could induce the activation of CXCL11/CXCR3 axis to exert pro-tumor effects in NPC.

In recent years, immune-checkpoint inhibitors (ICIs) targeting programmed death 1 (PD-1)/programmed death ligand 1 (PD-L1) have been used for antitumor therapy in multiple types of human cancers [9]. However, cancer cells can escape from immune attack through manipulating immune surveillance mechanisms to make immunotherapy ineffective. PD-1, a critical immune checkpoint receptor expressed on the surface of T cells, which can interact with its ligand PD-L1 expressed on tumor cell surface. This interaction delivers inhibitory signals that suppress T cell activation, ultimately enabling tumor immune evasion. Elevated PD-L1 expression has been observed in various malignancies, including NPC [10]. Researches have demonstrated that PD-L1 expression is controlled through multiple regulatory levels, including transcriptional regulation. As revealed by previous studies, CD274 (PD-L1 mRNA) can be transcriptionally activated by its upstream transcription regulators to promote immune evasion in human malignancies [11,12]. According to a previous report, CXCL11 could influence the expression and infiltration CD274 in oral squamous cell carcinoma [13]. Additionally, it has been documented in the literature that CXCR3 positively regulates NF-κB pathway activity [14]. CXCR3 has three ligands, including CXCL9, CXCL10, and CXCL11. According to previous studies, the CXCL10/CXCR3 axis is closely associated with NF-κB pathway activity [15]. Moreover, activation of the NF-κB pathway has been shown to upregulate PD-L1 expression [16] in tumor cells. Inhibition of p65 expression can lead to downregulation of PD-L1 [17]. However, whether the CXCL11/CXCR3 axis influences the nuclear translocation of p65 to drive PD-L1 transcriptional activation remains unknown.

To summarize, this work mainly aims to elucidate how CAFs-derived CXCL11 contributes to NPC malignancy and investigate the specific mechanism through which CXCL11/CXCR3 axis regulates the transcriptional activity of PD-L1 to mediate this process.

## Materials and methods

### Collection of tissue samples

This study was conducted in accordance with the Declaration of Helsinki and received approval from the Ethics Committee of the First Affiliated Hospital of Zhengzhou University. All participating patients provided written informed consent prior to inclusion. Paired nasopharyngeal carcinoma (NPC) tumor specimens and adjacent non-cancerous tissues were surgically resected from patients undergoing treatment at the Department of Head and Neck Surgery of our institution.

### Isolation and identification of CAFs and normal fibroblasts (NFs)

Primary cell cultures were established from fresh NPC and adjacent normal tissues. Briefly, the tissue specimens were first rinsed in serum-free medium to remove debris. Subsequently, the tissues were mechanically minced into explants approximately 0.2 × 0.2 mm in size and transferred to culture dishes containing appropriate growth medium. Following a 24 h incubation period, non-adherent cells were discarded by washing, and the remaining adherent cell population was cultured for an additional 2–3 weeks to allow for expansion. The CAFs were identified by detecting the fibroblast marker α-SMA using immunofluorescence (IF) staining. CAFs and NFs refer to fibroblasts isolated from tumor and non-tumor tissues, respectively.

### Cell culture

The NPC cell lines C666-1, HK-1, and CNE-3 were obtained from the Cell Bank of the Chinese Academy of Sciences (Shanghai, China) and Shanghai Fuheng Biotechnology Co., LTD (Shanghai, China), respectively. These NPC lines were cultured in RPMI-1640 medium (Gibco). The normal nasopharyngeal epithelial cell line, NP69, was purchased from Wuhan Procell Life Science and Technology Co., LTD (Wuhan, China) and cultured in its recommended medium (Procell). Primary CAFs and NFs were maintained in DMEM (Gibco). All media were supplemented with 10% fetal bovine serum (FBS; HyClone) and 1% penicillin-streptomycin (P/S; Solarbio). All cell lines were maintained in a humidified incubator at 37°C with 5% CO_2_.

### IF staining

For immunofluorescence analysis, cells were fixed with 4% paraformaldehyde, permeabilized with 0.1% Triton X-100 in PBS, and blocked with 10% bovine serum albumin (BSA) in PBS. Subsequently, the cells were incubated overnight at 4°C with primary antibodies targeting p65 (1:250 dilution; Proteintech) or α-SMA (1:50 dilution; Proteintech). Following PBS washes, samples were incubated with an FITC-conjugated secondary antibody (1:1000; Proteintech) for 1 h at 37°C in the dark. Nuclei were counterstained with 4,’6-diamidino-2-phenylindole (DAPI). Images were captured using a fluorescence microscope (Nikon, Japan).

### Cell transfection

To achieve CXCL11 knockdown, short hairpin RNAs (shRNAs) specifically targeting CXCL11 and a non-targeting control shRNA (sh-NC) were custom-synthesized by RiboBio (Guangzhou, China). For p65 overexpression, the full-length coding sequence of human p65 was cloned into the pcDNA3.1 expression vector (GenePharma, Shanghai, China), hereafter referred to as pcDNA-p65. All transient transfections of these constructs were performed using the Lipofectamine 2000 reagent (Thermo Fisher Scientific) according to the manufacturer’s protocol. Briefly, cells were seeded in 6-well plates and cultured until they reached 70–90% confluence at the time of transfection. Plasmids (2.5 μg) and the Lipofectamine 2000 reagent (6 μL) were separately diluted in DMEM (150 μL) to ensure optimal dispersion. Following a 5-min equilibration, the diluted plasmid was combined with the diluted Lipofectamine 2000 (1:1 ratio) and incubated at room temperature for 20 min. The resulting complexes were then added dropwise to the culture medium. To minimize potential liposome-induced cytotoxicity, the transfection medium was replaced with fresh complete growth medium after 4 h of incubation. Subsequent experiments were conducted 24 h post-transfection.

### RT-qPCR

Trizol reagent was applied for total RNA extraction. cDNA was obtained by performing reverse transcription using the SureScript™ First-Strand cDNA Synthesis Kit (Guangzhou iGene Biotechnology Co., Ltd., Guangzhou, China). Then, RT-qPCR was completed on an Applied Biosystems 7500 real-time PCR system (Thermo Fisher Scientific Inc.) by applying the TB Green Premix Ex Taq™ II kit (Takara, Dalian, China). The 2^−ΔΔCt^ calculation method was applied for the quantification of relative gene expression with β-actin as the internal reference gene.

### Western blot

RIPA buffer (Beyotime, Shanghai, China) was used to obtain the cell lysates and extract the total protein. Protein samples were subjected to electrophoresis, followed by transferring onto the membranes. After blocking with the nonfat milk, the membranes were incubated with primary antibodies: anti-CXCL11 (1:1,000 dilution; Abcam, Cambridge, CA, USA), anti-CXCR3 (1:1,000 dilution; Abcam), and the internal control anti-β-actin (1:1,000 dilution; HUABIO, Hangzhou, China) at 4°C overnight. Afterward, the membranes were subjected to further incubation with HRP-conjugated secondary antibodies (1:2,000 dilution, HUABIO). To visualize protein bands, membranes were treated using an ECL kit (Beyotime). Finally, the ImageJ software was used for the quantification of protein intensity in each sample.

### CCK-8 assay

After indicated treatments or transfections, 2,000 NPC cells were inoculated into each well of 96-well plates. At the beginning of the 2-h incubation, 10 μL of CCK-8 reagent (Beyotime) added into each well. After incubation, the OD value at 450 nm was measured at different time points by using a microplate reader.

### Cell proliferation assay

Following treatments, cells were incubated with 10 μM EdU for 2 h to label newly synthesized DNA. Subsequently, cells were fixed with 4% paraformaldehyde, permeabilized with 0.5% Triton X-100, and the incorporated EdU was visualized via a copper-catalyzed click reaction according to the manufacturer’s protocol. Cell nuclei were counterstained with DAPI. Images were captured using a fluorescence microscope, and the proliferation rate was determined by calculating the percentage of EdU-positive nuclei relative to the total number of DAPI-stained nuclei.

### Transwell invasion assays

Briefly, NPC cells were cultured in serum-free medium in the upper chamber of the transwell chamber pre-coated with Matrigel (Corning), while DMEM containing 10% FBS was added into the lower chamber. Twenty-four hours later, cells adhered to the lower surface of the upper chamber were treated with 4% paraformaldehyde (Beyotime) for fixation, and stained by 0.5% crystal violet dye for 10 min for visualization. The number of invaded cells was monitored and calculated under a light microscope.

### Wound healing assay

The migratory capacity of NPC cells was evaluated using a wound-healing assay. Cells were seeded into 6-well plates and grown to 80%–90% confluency. To inhibit cell proliferation and ensure that wound closure was due to cell migration, the monolayers were pre-treated with 10 μg/mL mitomycin C (Selleck Chemicals) for 1 h. A linear scratch was then created across the center of each monolayer using a sterile 200 μL pipette tip. After gently washing with PBS to remove detached cells, the cells were cultured in serum-free medium. The wound gap was imaged immediately (0 h) and at 24 h post-scratch using a light microscope. Wound closure was quantified by measuring the change in the wound area over time using ImageJ software.

### Co-immunoprecipitation (co-IP) assay

The interaction between CXCR3 and CXCL11 was investigated by Co-IP. Cells cultured in T75 flasks were harvested and lysed on ice using a triton-based lysis buffer. The resulting cell lysates were clarified by centrifugation at 12,000 × g for 10 min at 4°C. To reduce nonspecific binding, the supernatant was pre-cleared by incubation with Protein A/G PLUS-Agarose beads (Santa Cruz) for 1 h. A portion of the pre-cleared lysate was then incubated overnight at 4°C with either anti-CXCR3 antibody (1:200; Santa Cruz) or anti-CXCL11 antibody (1:30; Abcam). Subsequently, fresh Protein A/G PLUS-Agarose beads were added to capture the immunocomplexes. The beads were washed three times with lysis buffer, and the bound proteins were eluted by boiling in 2× SDS-PAGE loading buffer. The co-immunoprecipitated proteins were then detected by western blot analysis.

### Luciferase reporter assay

CD274 promoter sequences containing the p65 binding site or the mutant binding site were inserted into the pGL3-Basic vector (Promega Corporation, Madison, WI, USA) to construct the wild type or mutant type luciferase reporter plasmid (named WT or MUT). The reporter plasmids were transfected into NPC cells along with empty pcDNA3.1 vector or p65 expression vector using Lipofectamine 2000 (Thermo Fisher Scientific Inc.). The luciferase activity was detected by applying a dual luciferase assay system (Promega Corporation) at 48 h after co-transfections.

### Animal experiments

Animal experiments received approval and guidance from the Ethics Committee of the First Affiliated Hospital of Zhengzhou University. Health male BALB/c nude mice (four-week-old, *n* = 20) were purchased from Shanghai GemPharmatech. Housing conditions were controlled at a temperature of 25 ± 2°C and 60 ± 5% humidity, with a 12-h light/dark cycle. All animals had free access to food and water. Using a simple randomization method, mice were randomly assigned to four experimental groups, including Control, CAFs, CAFs/sh-NC, and CAFs/sh-CXCL11 groups (*n* = 5/each group). Next, mice were subcutaneously injected with 5 × 10^5^ HK-1 cells alone (Control) or co-injected with 5 × 10^5^ CAFs (CAFs) or 5 × 10^5^ CAFs transfected with sh-NC (CAFs/sh-NC) or sh-CXCL11 (CAFs/sh-CXCL11). Tumor growth was monitored and recorded every 7 days and then calculated in accordance with the following formula: 0.5×width^2^×length. After five weeks of subcutaneous implantation, mice were sacrificed by cervical dislocation, and the tumors in each group were separately collected and weighed for subsequent detections, which were conducted by an investigator blinded to the group allocation. Tumor samples in four different groups were then isolated for RT-qPCR or immunohistochemistry (IHC) analyses as mentioned before [18].

### Statistical analysis

Data from three independent experiments were analyzed and were plotted as means ± standard deviation (SD) by using Graphpad Prism 9 (Version 9.4.0). The normality was checked using the Shapiro – Wilk test. The equality of variances was evaluated with F test (two groups) or Brown – Forsythe test (multiple groups). Student’s *t*-tests were used for two-group comparisons. Multiple-group comparisons were conducted by using one-way/two-way ANOVA followed by Tukey’s multiple comparison test. Data with *p* value less than 0.05 were defined to be statistically significant.

## Results

### CXCL11 is highly expressed in CAFs and NPC cells

**Figure 1.**
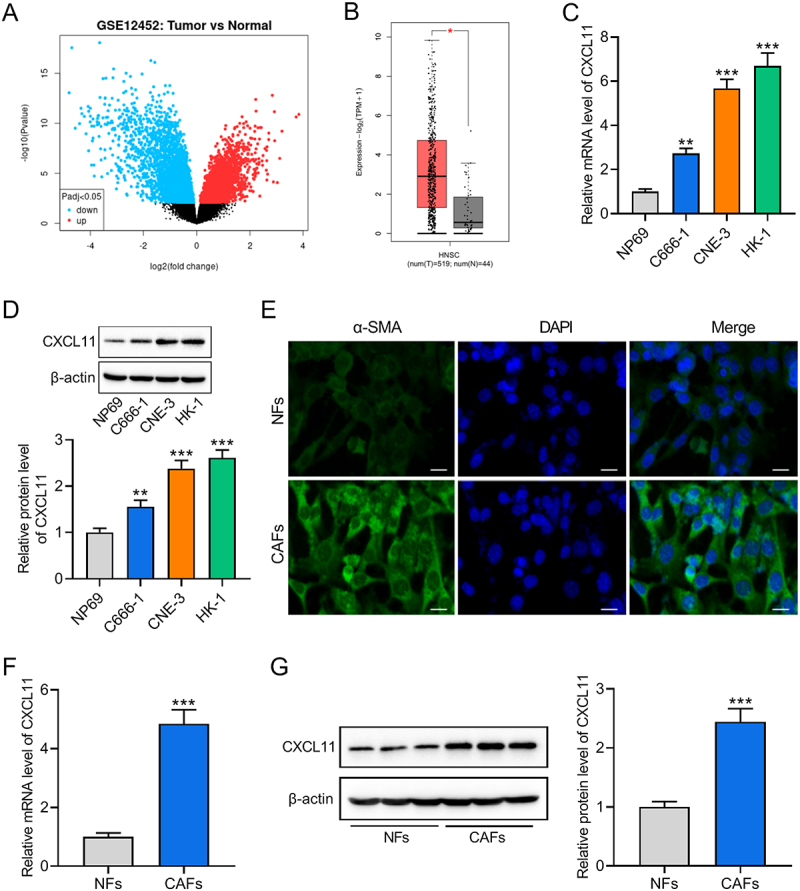
CXCL11 is highly expressed in CAFs and NPC cells. (A) the most significantly upregulated gene in tumor tissues obtained from NPC patients was identified by analyzing the GEO dataset GSE12452. (B) data obtained from GEPIA database revealed the CXCL11 expression in HNSC tumor tissues and normal tissues. (C, D) CXCL11 mRNA and protein levels were determined in NPC cell lines (C666-1, CNE-3, HK-1) and the normal nasopharyngeal epithelial cell line NP69 using RT-qPCR (C) and western blot (D). **p < .01, ***p < .001 vs. NP-69. (E) IF staining was applied to detect the expression of α-SMA in CAFs and NFs. Scale bar: 20 μm. (F G) CXCL11 mRNA and protein levels were measured in CAFs and NFs by using RT-qPCR (F) and western blot (G) Data were expressed as the mean ± SD of three independent biological replicates. ***p < .001 vs. NFs.

We first screened and analyzed the GEO dataset GSE12452, identifying CXCL11 as the most significantly upregulated gene in tumor tissues obtained from NPC patients (Figure 1(A)). Further analysis of the GEPIA database (http://gepia2.cancer-pku.cn/#analysis) confirmed that CXCL11 was markedly elevated in tumor tissues from head and neck squamous cell carcinoma (HNSC) patients compared to normal tissues (Figure 1(B)). To further validate the dysregulation of CXCL11 in NPC, we measured CXCL11 expression in NPC cell lines (C666-1, CNE-3, HK-1) and NP69 cells using RT-qPCR and western blot. Results showed that both mRNA and protein levels of CXCL11 were significantly higher in NPC cells than those in NP69 cells (Figure 1(C, D)). Since CAFs secrete chemokines (including CXCL family members) to promote tumor progression [4,19], we further examined CXCL11 expression in CAFs. Before that, we isolated CAFs from NPC tumor tissues and NFs from non-cancerous tissues. Expression of the CAF marker α-SMA was higher in CAFs than that in NFs (Figure 1(E)). Subsequent analyses unveiled that CXCL11 mRNA and protein levels were significantly higher in CAFs than in NFs (Figure 1(F, G)). These findings suggest that CXCL11 May be secreted by CAFs into NPC cells, contributing to its aberrant expression in NPC.

### CAFs-secreted CXCL11 promotes NPC cell proliferation, invasion, and migration

**Figure 2.**
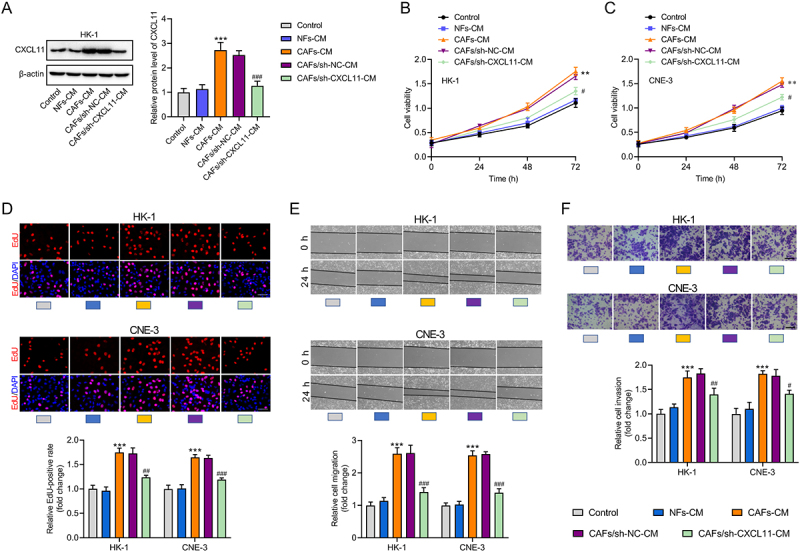
CAFs-secreted CXCL11 promotes NPC cell proliferation, invasion, and migration. (A) CXCL11 protein levels were measured in HK-1 cells cultured in conditioned medium from NFs, CAFs, CAFs/sh-NC, and CAFs/sh-CXCL11 by western blot. ***p < .001 vs. NFs-CM; ^###^*p* < .001 vs. CAFs/sh-NC-CM. (B, C) the viability of NPC cells cultured in conditioned medium from NFs, CAFs, CAFs/sh-NC, and CAFs/sh-CXCL11 was evaluated by CCK-8 assays. **p < .01 vs. NFs-CM; ^#^*p* < .05 vs. CAFs/sh-NC-CM. (D) EdU assays were conducted to determine the proliferation ability of NPC cells cultured in conditioned medium from NFs, CAFs, CAFs/sh-NC, and CAFs/sh-CXCL11. Scale bar: 50 μm. ***p < .001 vs. NFs-CM; ^##^*p* < .01, ^###^*p* < .001 vs. CAFs/sh-NC-CM. (E, F) the migratory and invasive capacities of NPC cells under different culture conditions were assessed by using wound healing (E) and transwell (F) assays. Scale bar: 50 μm. Data were expressed as the mean ± sd of three independent biological replicates. ***p < .001 vs. NFs-CM; ^#^*p* < .05, ^##^*p* < .01, ^###^*p* < .001 vs. CAFs/sh-NC-CM.

Given that CAFs-secreted chemokines enhance malignant behaviors of tumor cells [20,21], we investigated whether CAF-secreted CXCL11 influences NPC cell proliferation, invasion, and migration. To prove the extracellular secretion of CXCL11 by CAFs, CAFs were treated with the protein secretion inhibitor brefeldin A (an inhibitor of ER-Golgi trafficking, BFA) for 24 h. IF staining demonstrated that CXCL11 levels were elevated in CAFs following BFA treatment (Figure S1). NPC cells were cultured in conditioned medium (CM) from NFs, CAFs, CAFs transfected with sh-NC, and CAFs transfected with sh-CXCL11. Through western blot, we confirmed that CXCL11 levels were unchanged in NPC cells cultured with NFs-CM but significantly increased in CAFs-CM. Knockdown of CXCL11 in CAFs reduced its expression in NPC cells (Figure 2(A)). Functionally, the viability of NPC cells cultured with CAFs-CM was strengthened, which was reduced again after silencing of CXCL11 in CAFs (Figure 2(B, C)). According to EdU data, NPC cells cultured with CAFs-CM had a higher proliferation ability than those cultured with NFs-CM, whereas knockdown of CXCL11 in CAFs led to the decrease of proliferation ability of cultured NPC cells (Figure 2(D)). As depicted in Figure 2(E,F), exposure to CAFs-CM resulted in a significant increase in the migratory and invasive potential of NPC cells compared to those cultured in NFs-CM. Crucially, this pro-malignant effect was substantially attenuated when NPC cells were cultured with CM from CAFs in which CXCL11 had been silenced. These findings collectively demonstrate that CXCL11 secreted by CAFs is a key mediator of NPC cell migration and invasion.

### CAFs-derived CXCL11 upregulates CXCR3 in NPC cells

**Figure 3.**
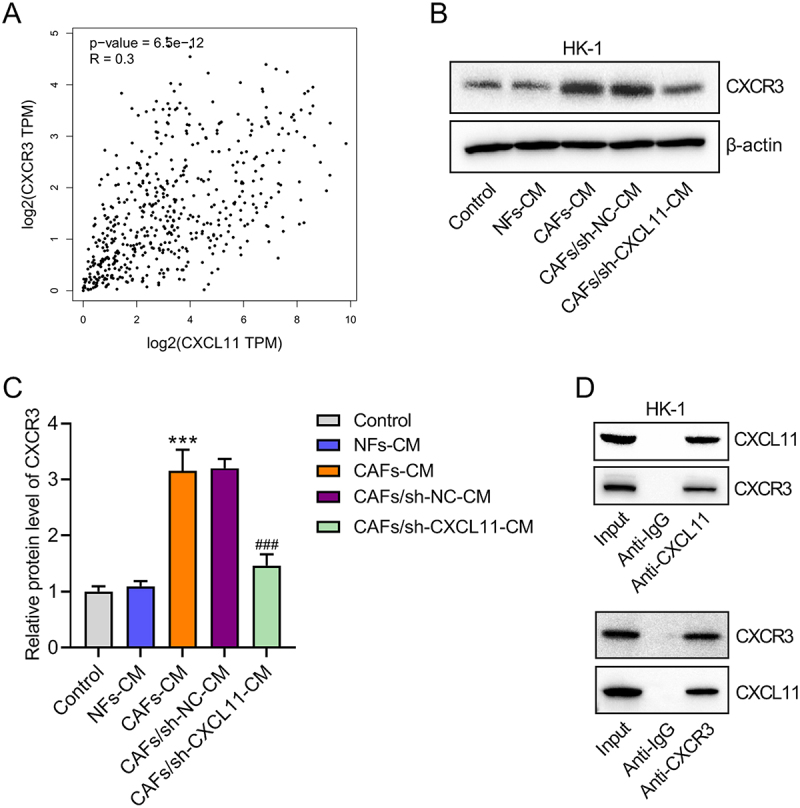
CAFs-derived CXCL11 upregulates CXCR3 in NPC cells. (A) the positive correlation between CXCL11 and CXCR3 expression in HNSC tumor tissues was analyzed based on data from GEPIA database. (B, C) the effect of CAFs-derived CXCL11 on CXCR3 protein expression in NPC cells was assessed by western blot analysis. (D) Co-IP assay was conducted to validate CXCL11-CXCR3 interaction in NPC cells. Data were expressed as the mean ± sd of three independent biological replicates. ***p < .001 vs. NFs-CM; ^###^*p* < .001 vs. CAFs/sh-NC-CM.

CXCR3 is a receptor for the chemokine CXCL11. In an immunosuppressive microenvironment, CXCL11 could bind to CXCR3 to activate tumor-associated signaling pathways and contribute to tumor progression. This study further investigates whether CAF-derived CXCL11 can activate CXCR3 and exert pro-tumor effects in NPC. First, data retrieved from the GEPIA database showed a positive correlation between CXCL11 and CXCR3 expression in HNSC tumor tissues (Figure 3(A)). Subsequently, we examined the effect of CAFs-derived CXCL11 on CXCR3 protein expression in NPC cells. Western blot results indicated that compared with the control group, CXCR3 protein levels remained unchanged in NPC cells cultured with NFs-CM, but were significantly increased in NPC cells cultured with CAFs-CM. However, culture with conditioned medium from CXCL11-knockdown CAFs reduced CXCR3 protein expression in NPC cells (Figure 3(B,C)). Furthermore, co-IP assay demonstrated that CXCL11 and CXCR3 interact in NPC cells (Figure 3(D)). Therefore, we confirm that CXCL11-secreted by CAFs upregulates CXCR3 in NPC cells.

### CAFs-secreted CXCL11 upregulates CXCR3 expression to facilitate NPC cell proliferation, migration, and invasion

**Figure 4.**
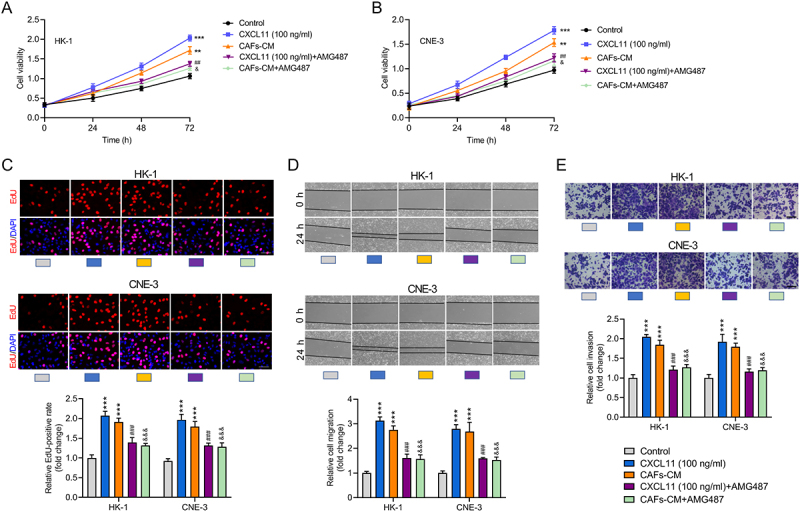
CAFs-secreted CXCL11 induces CXCR3 activation to facilitate the malignant progression of NPC. (A, B) The effects of CXCL11/CXCR3 axis on the viability of NPC cells were detected by performing CCK-8 assays. (C) The proliferation ability of NPC cells treated with CXCL11, CAFs-CM, or with additional treatment of AMG487 was evaluated by EdU assays. (D) Wound healing assays were carried out for detecting the migration ability of NPC cells treated with CXCL11, CAFs-CM, or with additional treatment of AMG487. (E) Transwell assays were carried out for detecting the invasive ability of NPC cells treated with CXCL11, CAFs-CM, or with additional treatment of AMG487. Data were expressed as the mean ± SD of three independent biological replicates. **p < .01, ***p < .001 vs. Control; ^##^*p* < .01, ^###^*p* < .001 vs. CXCL11 (100 ng/ml); ^&^*p* < .05, ^&&&^*p* < .001 vs. CAFs-CM.

To validate that CAFs-secreted CXCL11 promotes the malignant progression of NPC by upregulating the CXCR3 expression, we performed functional rescue experiments. CCK-8 assay results showed that the proliferation of NPC cells was significantly enhanced after treatment with CXCL11 and CAFs-CM; however, this enhancement was markedly attenuated upon addition of the CXCR3 antagonist AMG487 (Figure 4(A,B)). Similarly, EdU assay results indicated that NPC cell proliferation was significantly increased following CXCL11 and CAFs-CM treatment but was notably reduced after AMG487 administration (Figure 4(C)). Wound healing assays demonstrated that NPC cell migration ability was significantly enhanced by CXCL11 and CAFs-CM, whereas it was substantially inhibited in the presence of AMG487 (Figure 4(D)). Finally, transwell assay results revealed that the invasive capacity of NPC cells was significantly strengthened after CXCL11 and CAFs-CM treatment but was markedly weakened upon AMG487 addition (Figure 4(E)). These results collectively indicate that CAFs-derived CXCL11 upregulates CXCR3 expression in NPC cells, thereby promoting NPC cell proliferation, migration, and invasion.

### CXCL11/CXCR3 axis upregulates PD-L1 expression through p65-mediated transcription activation

**Figure 5.**
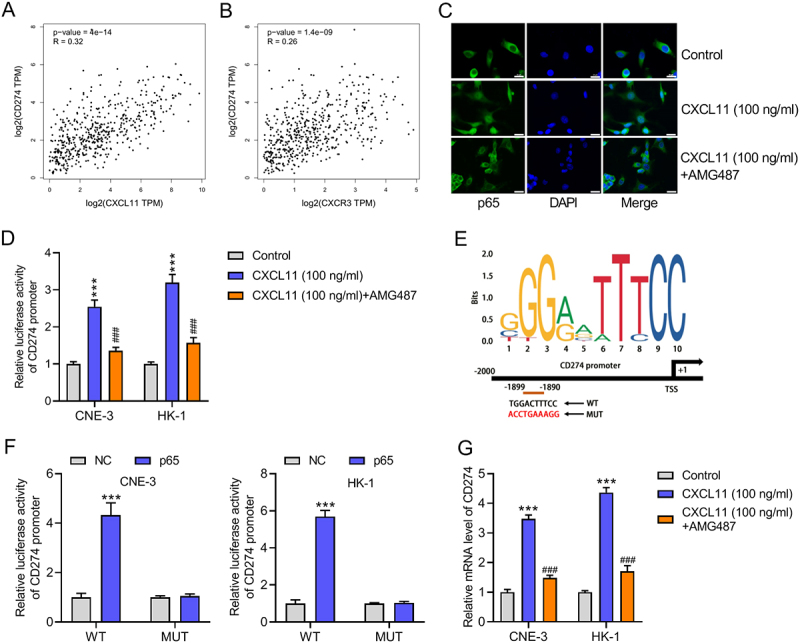
CXCL11/CXCR3 axis upregulates PD-L1 expression through p65-mediated transcription activation. (A, B) the expression correlation between CD274 (PD-L1 mRNA) and both CXCL11 and CXCR3 in HNSC tumor tissues was analyzed in accordance with data from GEPIA database. (C) IF staining was applied to detect the effect of CXCL11 and CXCR3 on p65 nuclear translocation. Scale bar: 20 μm. (D) Luciferase reporter assays were conducted to determine the effect of CXCL11/CXCR3 axis on the transcription activity of CD274. ***p < .001 vs. Control; ^###^*p* < .001 vs. CXCL11 (100 ng/ml). (E) The DNA motif of p65 and p65 binding site on the CD274 promoter were predicted using the JASPAR tool. (F) luciferase reporter assays were conducted to determine whether mutation of the binding site could abolish p65-mediated CD274 transcriptional activation. ***p < .001 vs. NC. (G) The effect of CXCL11 and inhibition of CXCR3 on CD274 mRNA levels in NPC cells was evaluated by RT-qPCR. Data were expressed as the mean ± SD of three independent biological replicates. ***p < .001 vs. Control; ^###^*p* < .001 vs. CXCL11 (100 ng/ml).

Given that the NF-κB signaling pathway, a known downstream effector of CXCR3, can induce the expression of PD-L1, we posited a specific mechanism for our observations. We hypothesized that the engagement of CXCR3 by its ligand, CXCL11, promotes the nuclear translocation of the NF-κB subunit p65. This event, in turn, would lead to the transcriptional upregulation of PD-L1, thereby contributing to the malignant progression of the tumor. According to GEPIA database analysis, CD274 (PD-L1 mRNA) expression showed a positive correlation with both CXCL11 and CXCR3 expression in HNSC tumor tissues (Figure 5(A, B)). IF results indicated that CXCL11 treatment increased nuclear translocation of p65 and reduced its cytoplasmic retention, whereas the addition of AMG487 attenuated this effect, suggesting that the CXCL11/CXCR3 axis promotes p65 nuclear entry (Figure 5(C)). Through luciferase reporter assays, we demonstrated that CXCL11 treatment significantly enhanced CD274 promoter activity, while AMG487 addition markedly reduced it (Figure 5(D)). To determine whether p65 directly regulates CD274 transcription, we first predicted p65 binding site on the CD274 promoter using JASPAR (https://jaspar.elixir.no/) (Figure 5(E)). Subsequent luciferase reporter assays showed that mutation of the binding site abolished p65-mediated CD274 transcriptional activation (Figure 5(F)), confirming the functional importance of the binding site. To confirm that the CXCL11/CXCR3 axis regulates PD-L1 at the transcriptional level, we performed RT-qPCR analysis. We found that stimulation with recombinant CXCL11 significantly increased the mRNA levels of CD274 (the gene encoding PD-L1). Conversely, pharmacological inhibition of CXCR3 with AMG487 resulted in a marked reduction in CD274 expression (Figure 5(G)). These results demonstrate that the CXCL11/CXCR3 axis directly promotes the transcription of CD274, providing a mechanism by which this pathway facilitates tumor growth.

### CAFs-secreted CXCL11 promotes NPC cell growth in vivo

**Figure 6.**
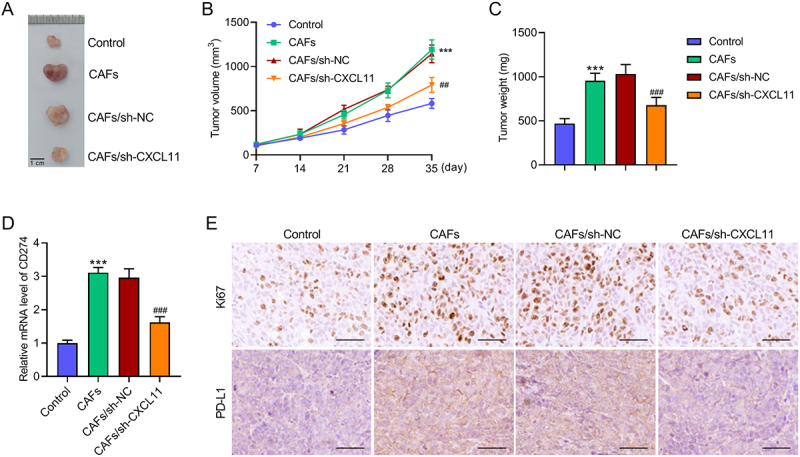
CAFs-secreted CXCL11 promotes NPC cell growth *in vivo*. (A) After five weeks of subcutaneous implantation, tumors were extracted from four groups of mice. Scale bar: 1 cm. (B) Tumor growth curves in four groups were recorded and shown. ***p < .001 vs. Control; ^##^*p* < .01 vs. CAFs/sh-NC. (C) Tumor weight in four groups. ***p < .001 vs. Control; ^###^*p* < .001 vs. CAFs/sh-NC. (D) RT-qPCR was used to measure CD274 expression in four groups. ***p < .001 vs. Control; ^###^*p* < .001 vs. CAFs/sh-NC. (E) IHC staining was performed to examine Ki67 and PD-L1 protein expression in four groups. Scale bar: 50 μm. Data were expressed as the mean ± SD of five independent biological replicates.

To validate our *in vitro* findings, we established xenograft mouse models by subcutaneously injecting HK-1 cells along, co-injected with CAFs, or co-injected with CAFs harboring a CXCL11 knockdown. Co-injection with CAFs resulted in a dramatic acceleration of tumor growth over a five-week period, this effect was suppressed when CXCL11 was silenced in the CAFs (Figure 6(A-C)). Tumors from the CAF co-injection group displayed elevated mRNA levels of CD274 (Figure 6(D)) and increased the protein levels of both Ki67 and PD-L1 (Figure 6(E)). Crucially, the expression of all these markers was restored to near-control levels in tumors containing CXCL11-deficient CAFs. Collectively, these *in vivo* data demonstrate that CAF-derived CXCL11 is a pivotal driver of NPC tumor progression and proliferation.

## Discussion

Immunotherapy using ICIs has emerged as a promising therapeutic approach for NPC, with PD-1/PD-L1 being the most clinically relevant targets [22]. By overcoming tumor immune evasion and reactivating intrinsic anti-tumor immune responses, ICIs represent a breakthrough in cancer treatment. Our investigation provides novel evidence that targeting CXCL11 could suppress NPC progression by negatively regulating PD-L1 expression, offering potential strategies to improve immunotherapy outcomes through mechanistic understanding of PD-L1 modulation.

CXCL11 has been identified as a metastasis predicator and a novel biomarker in colorectal cancer [23]. Moreover, inhibiting CXCL11 secreted by CAFs could effectively suppress colorectal cancer progression [24]. This study first revealed the role of CXCL11 in mediating NPC progression. Our investigation began with bioinformatics analysis, which revealed that the chemokine CXCL11 is significantly upregulated in NPC and HNSC. We validated this finding at the cellular level, confirming elevated CXCL11 expression in NPC cells. Given that CAFs-secreted chemokines enhance malignant behaviors of tumor cells [20,21], we examined CXCL11 expression in CAFs and confirmed the upregulation of CXCL11 in CAFs. Based on these observations, we hypothesized that CAF-derived CXCL11 plays a key role in the tumor microenvironment. To test this, we cultured NPC cells with CAFs-CM, which markedly enhanced their proliferative, migratory, and invasive capacities compared to controls. Crucially, this pro-tumorigenic effect was significantly attenuated when CXCL11 was silenced in the CAFs. These findings collectively demonstrate that CXCL11 secreted by CAFs is a critical mediator of NPC progression, promoting key malignant phenotypes.

CXCR3 is a receptor for the chemokine CXCL11. Increased secretion of CXCL11 from donor cells can contribute to the elevation of CXCR3 expression in recipient cells [7]. In an immunosuppressive microenvironment, the CXCL11/CXCR3 axis has been proven to be the activator of tumor-associated signaling pathways in during tumor progression [8]. Here, we identified the positive regulatory effect of CAFs-derived CXCL11 on CXCR3 expression in NPC cells. Moreover, CXCL11 could interact with CXCR3 in NPC cells. According to functional rescue experiments, the proliferative, migratory, and invasive capacities of NPC cells enhanced by the treatment with CXCL11 and CAFs-CM were markedly suppressed upon addition of the CXCR3 antagonist AMG487. These results demonstrated that CAF-derived CXCL11 upregulated CXCR3 expression to exert pro-tumor effects in NPC.

Dysregulation of PD-L1 has been detected in various human malignancies such as colorectal cancer [25], lung cancer [26], and NPC [27]. Upregulation of PD-L1 in human cancers could induce immune escape and accelerate tumor progression in various human cancers, including NPC. NPC cell-derived exosomal PD-L1 exacerbates CD8^+^ T cell suppression to promote NPC tumor growth [28]. Beyond its immunoregulatory role, PD-L1 contributes to tumorigenesis by influencing cancer cell proliferation, apoptotic resistance, migration, and invasion. For example, PD-L1 overexpression promotes the migratory and invasive capacities of head and neck cancer cells [29]. APLNR inhibits NPC growth through suppression of PD-L1 expression [30]. Downregulation of PD-L1 expression by nano-coated si-SNHG14 restrains epithelial-mesenchymal transition in NPC cells [31]. The current study unveiled the functions of CXCL11/CXCR3 in PD-L1-mediated.

Previous mechanism investigations have revealed that PD-L1 can be upregulated by transcriptional modification such as transcription activation and promoter methylation regulation [32,33]. In this study, we also unmasked the transcriptional regulation of PD-L1 mediated by CXCL11 in NPC. Studies have shown that protein-protein interactions can facilitate nuclear translocation of transcription factors to activate downstream targets [22,23]. Here, we demonstrated that CXCL11 interacted with the transcription factor p65. It has been widely reported that p65 could be regulated by its upstream protein to translocate into cell nucleus and thus play the role of transcriptional activation [34,35]. CXCR3 could interact with its ligands such as CXCL10 to increase NF-κB pathway activity [15]. The activation of the NF-κB pathway is a well-established mechanism for upregulating PD-L1 expression in tumor cells [16]. The current study revealed that CAFs-derived CXCL11 could cooperate with CXCR3 to promote the nuclear translocation of p65 and thus transcriptionally activate PD-L1 to facilitate malignant phenotype of NPC cells. Through IF analysis, we proved that CXCL11 promoted p65 nuclear accumulation. Further luciferase reporter assays verified that p65 could bind to CD274 promoter to transcriptionally activate CD274 and enhance PD-L1 expression. Therefore, we confirmed that CXCL11 promoted the nuclear translocation of p65 to transcriptionally activate CD274 in NPC cells.

**Figure 7.**
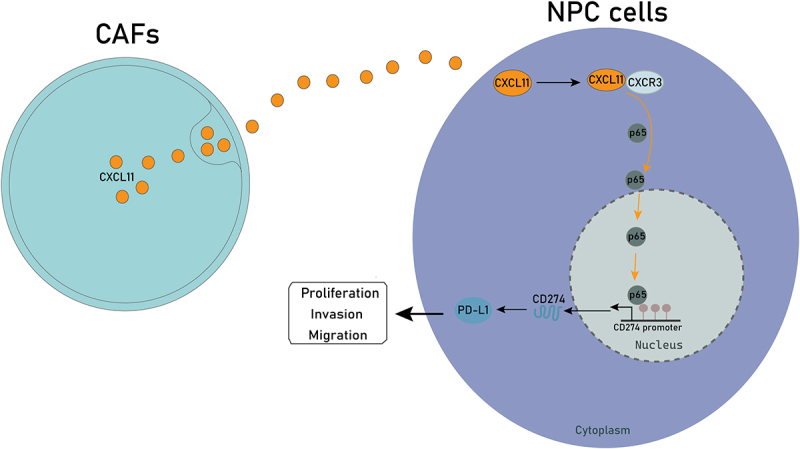
Schematic illustration of CAFs-secreted CXCL11 promoting NPC progression. CAFs-derived CXCL11 activates CXCL11/CXCR3 axis in NPC cells to promote p65-mediated PD-L1 transcription activation, thereby promoting the malignant phenotypes of NPC cells.

In conclusion, we verified that CAFs secrete CXCL11 to promote the malignant phenotype of NPC cells. Mechanistically, we revealed that CXCL11/CXCR3 axis upregulated PD-L1 expression through p65-mediated transcription activation (Figure 7). All our findings indicated that targeting CXCL11 might be conducive to enhancing the efficacy of immunotherapy for NPC patients.

## Supplementary Material

Supplemental Material

## Data Availability

The data that support the findings of this study are available from the corresponding author upon reasonable request.
