# Supplementary figures and images for: CXCL11 secreted by cancer-associated fibroblasts promotes nasopharyngeal carcinoma progression via CXCR3/PD-L1 axis
Source: Cell Adh Migr. 2026 May 4;20(1):2665498. doi: 10.1080/19336918.2026.2665498 (PMC13155017; doi:10.1080/19336918.2026.2665498)

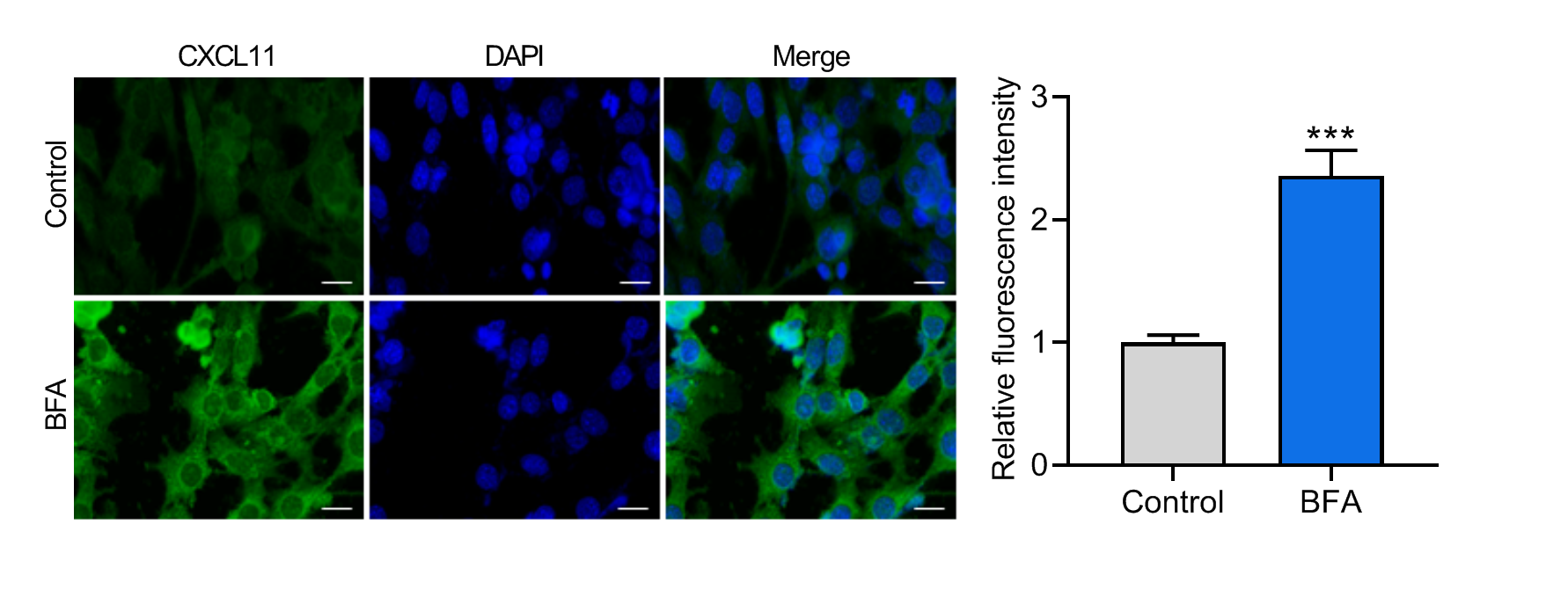

Supplement: Supplemental Material [file KCAM_A_2665498_SM9655.tif]
